# Specific Mode Electroacupuncture Stimulation Mediates the Delivery of NGF Across the Hippocampus Blood–Brain Barrier Through p65-VEGFA-TJs to Improve the Cognitive Function of MCAO/R Convalescent Rats

**DOI:** 10.1007/s12035-024-04337-8

**Published:** 2024-07-12

**Authors:** Mengyuan Dai, Kecheng Qian, Qinyu Ye, Jinding Yang, Lin Gan, Zhaoxing Jia, Zixing Pan, Qian Cai, Tianxiang Jiang, Congcong Ma, Xianming Lin

**Affiliations:** 1 The Third Clinical Medical College, Zhejiang Chinese Medical University, 548 Binwen Road, Binjiang District, Hangzhou, 310051 Zhejiang Province China; 2 Key Laboratory of Acupuncture and Neurology of Zhejiang Province, Hangzhou, China; 3 Department of Rehabilitation, Lishui Central Hospital, Lishui, 323000 Zhejiang Province China; 4 The Third Affiliated Hospital of Zhejiang, Chinese Medical University, Xihu District, Moganshan Road No. 219, Hangzhou, 310000 Zhejiang Province China; 5 Department of Rehabilitation, Zhejiang Rehabilitation Medical Center, No. 2828, Binsheng Road, Hangzhou, 310051 Zhejiang Province China

**Keywords:** Ischemic stroke, Cognitive dysfunction, Nerve growth factor (NGF), Electroacupuncture stimulation, Blood–brain barrier (BBB), Cholinergic neurons

## Abstract

Cognitive impairment frequently presents as a prevalent consequence following stroke, imposing significant burdens on patients, families, and society. The objective of this study was to assess the effectiveness and underlying mechanism of nerve growth factor (NGF) in treating post-stroke cognitive dysfunction in rats with cerebral ischemia–reperfusion injury (MCAO/R) through delivery into the brain using specific mode electroacupuncture stimulation (SMES). From the 28th day after modeling, the rats were treated with NGF mediated by SMES, and the cognitive function of the rats was observed after treatment. Learning and memory ability were evaluated using behavioral tests. The impact of SMES on blood–brain barrier (BBB) permeability, the underlying mechanism of cognitive enhancement in rats with MCAO/R, including transmission electron microscopy, enzyme-linked immunosorbent assay, immunohistochemistry, immunofluorescence, and TUNEL staining. We reported that SMES demonstrates a safe and efficient ability to open the BBB during the cerebral ischemia repair phase, facilitating the delivery of NGF to the brain by the p65-VEGFA-TJs pathway.

**Graphical Abstract:**

By Figdraw

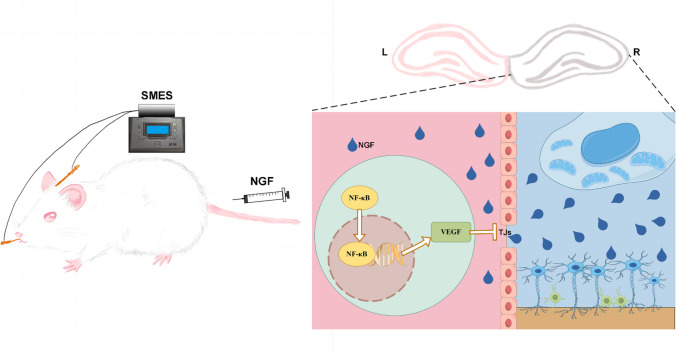

**Supplementary Information:**

The online version contains supplementary material available at 10.1007/s12035-024-04337-8.

## Introduction

Ischemic stroke is a prominent etiological factor for cognitive impairment on a global scale [1, 2]. Post-stroke cognitive impairment (PSCI) encompasses a range of syndromes that satisfy the diagnostic criteria for cognitive impairment occurring within 6 months following a stroke event [3]. This definition underscores the significance of the potential causal association between stroke and cognitive impairment, as well as the interrelation between clinical management and cognitive decline. The emergence of research findings on PSCI has propelled it to the forefront of contemporary international stroke research and intervention [4–6]. Cognitive impairment frequently presents as a prevalent consequence of stroke, imposing significant burdens on patients, families, and society [7]. Regrettably, this issue has historically been overlooked. Stroke not only predisposes patients to cognitive dysfunction but also exacerbates its progression, potentially culminating in dementia [8]. Research findings indicate that stroke amplifies the likelihood of dementia in patients by a factor of 4 to 12 [9].

Research has demonstrated that ischemic stroke elicits impairment of cholinergic neurons via the mechanisms of endoplasmic reticulum stress, oxidative stress, and inflammatory response [10, 11]. The cholinergic system, which employs acetylcholine as a neurotransmitter, is closely linked to cognitive processes such as memory and selective attention [12]. Consequently, the cholinergic pathway and nervous system are susceptible to vascular injury, ultimately resulting in PSCI [13].

Nerve growth factor (NGF) exerts its primary effects on basal forebrain cholinergic neurons (BFCN) upon crossing the blood–brain barrier (BBB) [14, 15]. NGF assumes a crucial role in facilitating the growth, repair, and survival of BFCN, thereby mitigating damage to the cholinergic system and minimizing its consequences [16, 17]. The basal forebrain cholinergic projection is accountable for the synthesis of neurotrophic factors in both the cortical target region and the cholinergic cell soma region [18]. The primary obstacle lies in the macromolecular nature of NGF, which possesses a molecular weight of 13.4 KD, rendering it arduous to traverse the blood–brain barrier and attain optimal concentrations within the central nervous system [19]. Consequently, the utilization of NGF in central nervous system disorders is constrained. Consequently, a significant area of emphasis in current neurorehabilitation research revolves around devising approaches to expedite the transportation of NGF to the cerebral tissue, thereby achieving efficacious drug concentrations in the bloodstream that facilitate its involvement in augmenting neurotrophic and cortical plasticity. Currently, the methods employed for opening the BBB encompass the following approaches: the application of hypertonic solutions, such as mannitol; the utilization of vasoactive substances like histamine and bradykinin; the use of ultrasonic and electromagnetic fields; the manipulation of transporters, such as liposomes; the regulation of the BBB drug efflux system; the administration of traditional Chinese medicine, including borneol, musk, *Acorus tatarinowii*, and other resuscitation drugs; nasal feeding or lateral ventricle injection; the combination of drugs with vectors such as viruses, nanoparticles, and exosomes; and the utilization of peptides and ABC transporter inhibitors [20–22]. Numerous challenges exist associated with the abovementioned methods of opening the BBB, particularly in addressing complications such as brain edema and significant neuronal apoptosis induced by the procedure. Additionally, each method is constrained by its specific techniques and technological limitations, thereby presenting a considerable gap in achieving practical implementation within clinical settings [23, 24]. The previous study conducted by our research group revealed that the application of electroacupuncture at the Shuigou and Baihui acupoints can effectively and safely induce the opening of the BBB in normal rats [25]. Based on the findings of the research group’s previous study, specific mode electroacupuncture stimulation (SMES) applied at Baihui and Shuigou acupoints has been shown to decrease the expression of tight junction proteins ZO-1 and occludin within the BBB. Additionally, SMES has been demonstrated to enhance the permeability of the BBB, allowing for the passage of Evans blue (EB-albumin, 68 kDa) and FITC-dextran (20 kDa) across the BBB. It is noteworthy that the molecular weights of these substances exceed that of NGF (13.4 kDa). This effect is most pronounced when utilizing density wave electroacupuncture with a frequency of 2/100 Hz, an intensity of 3 mA, and a stimulation pattern of 6 s, followed by 6 s off (6 s-6 s), repeated for 40 min [26–28].

We investigated the impact of SMES-induced NGF delivery on the enhancement of long-term memory and preservation of cholinergic neurons in rats subjected to MCAO/R to elucidate the mechanism by which SMES facilitates the transportation of NGF to the brain. The potential pathway for SMES-induced BBB permeability involves the p65-VEGFA-TJs pathway. This discovery offers novel insights into using macromolecular therapeutic agents to treat central nervous system disorders.

## Materials and Methods

### Animals

Adult male Sprague–Dawley rats weighing 230–250 g were obtained from the Animal Experimental Research Center of Zhejiang Chinese Medical University. The rats were housed under specific pathogen-free conditions for 1 week, with ad libitum access to food and water. The Animal Care and Use Committee of Zhejiang Chinese Medical University approved all animal experiments. All procedures were performed according to the National Institutes of Health Guidelines for Animal Use.

### Rat MCAO/R Model and Treatment

A rat model of middle cerebral artery occlusion and drug treatment was used in this study. Anesthesia was induced by intraperitoneal injection of 3% pentobarbital sodium at a dose of 30 mg/kg. The middle cerebral artery occlusion procedure, without craniectomy, was performed in rats according to established protocols [29].

Under sterile conditions, the right common carotid artery (CCA), external carotid artery (ECA), internal carotid artery (ICA), and junction were meticulously exposed and isolated through a midline incision. A silicone-coated suture (Beijing Xinong Technology Co., Ltd., Beijing, China) was introduced through the external carotid incision and advanced towards the initial point of the middle cerebral artery via the internal carotid artery. After 1.5 hours of ischemia, the suture was removed to restore cerebral blood flow. After 28 days of MCAO/R modeling, the rats were used for subsequent studies. Because at this time, the rats still had cognitive dysfunction, but due to the relatively complete recovery of the blood–brain barrier, macromolecular drugs could not enter the brain, so we selected it as a better intervention period for the study of SMES-mediated NGF into the brain [30, 31]. This time frame was chosen as the point at which the BBB on the infarct side essentially regained its functionality, thus enabling the opening of the BBB and the administration of macromolecular drugs through the SMES [32, 33].

Rats were administered mouse nerve growth factor (mNGF) (Haite Biology) dissolved in saline via tail vein injection 5 min before the experiment [34–36]. In the inhibitor group, pyrrolidine dithiocarbamate (PDTC) (150 mg/kg) (P-8765, Sigma, American) dissolved in saline was intraperitoneally injected 6 h before the initiation of SMES, which was performed according to the previously described protocol [26]. In comparison to conventional electroacupuncture, the specific mode of electroacupuncture stimulation involves the selection of a particular relay that alternates between 6 s of power on and 6 s off. Previous research conducted by our team demonstrated that this specific mode of electroacupuncture stimulation, utilizing parameters of 2/100 Hz, 3 mA, 40 min, and a 6-s on–off cycle, can effectively induce the opening of the blood–brain barrier through the stimulation of the GV26 (Shuigou) and GV20 (Baihui) acupoints [26, 27, 33]. A needle manufactured by Beijing Zhongyan Taihe Medical Instrument Co., Ltd., China, measuring 13 mm long and 0.25 mm in diameter, was inserted at the acupoint GV20. Another needle measuring 7 mm in length and 0.16 mm in diameter was inserted at acupoint GV26. Subsequently, an acupoint nerve stimulator (HANS-200; Nanjing Jinsheng Ltd., Nanjing, China) was connected. In the NGF permeation experiment and the p65-VEGFA-TJs mechanism study, we conducted a single intervention: the therapeutic effect of SMES-mediated NGF delivery across the BBB into the brain. We took a continuous daily treatment for 10 days.

### Neurological Scoring

An observer who was unaware of the treatment measured the neurological score 24 h after reperfusion in rats [37]: 0 were rats with no obvious defect behavior; 1, left anterior flexion of the rat tail when lifted; 2 indicate rats circling to the left; 3 denote rats leaning to the left; and 4 represented rats unable to walk autonomously, are comatose, or die. Only rats with scores of 1, 2, and 3 were used in subsequent experiments.

### Transmission *Electron* Microscopy

Transmission electron microscopy (TEM) was used to examine the mitochondrial morphological changes in the rat hippocampus. In short, the CA1 of the hippocampus was isolated, cut into 1 mm^2^ cubes, and fixed with 2.5% pentanediol in phosphate buffer and 1% osmium tetroxide. Following dehydration with alcohol, the samples were embedded in epoxy resin. Ultra-thin sections of 70 nm thickness were stained with lead citrate, and images were captured using an H-7650 TEM (Hitachi, Japan) to observe the morphological changes in the mitochondria.

### Morris Water Maze

The Morris water maze (MWM) test was used to detect the learning and memory abilities of MCAO/R rats after treatment. The diameter and depth of the water maze were 180 and 50 cm, respectively, and it was classified into four quadrants of equal size using fluorescent paper with different shapes. One quadrant had a circular platform (12 × 12 cm^2^) located 2 cm below the water surface. In the training experiment, the camera was positioned above the uppermost part of the maze. Each rat underwent four daily training sessions at 30-min intervals for 5 days. During each session, rats were allotted 60 s to locate the platform and permitted to remain there for 10 s. If the rat failed to locate the platform within the designated time frame, it was guided to the platform and allowed to stay there for 10 s. On the final day of the experiment, the platform was removed, and the frequency of platform crossings and the duration of residence in the target quadrant were recorded [38–40]. All experimental information was recorded using an ANY-maze system (Stoelting Co., American).

### Novel Object Recognition Test

On the 28th day after MCAO/R, a novel object exploration test (NOR) was conducted in a wooden box measuring 50 cm × 50 cm × 50 cm. The box floor was lined with gray paper. The rats were acclimated for 3 days before habituation. During this initial phase, the rats were placed in a box for 5 min without any objects present, and measurements were taken for central area exploration, exploration distance, and fecal particles. On the fourth day, two objects (A1 and A2) were positioned within the box, 10 cm away from the box wall, allowing the rats to explore freely for 5 min. Following a time-lapse of either 1 h or 24 h, object A2 was substituted with novel object B, enabling the rats to investigate the new object for 5 min freely. The duration spent identifying each object was documented using the ANY-maze system [41, 42]. The index (B-A1)/(B + A1) was employed to quantify the ratio between the temporal disparity in exploring familiar and novel objects and the overall duration of exploring both objects. This index serves as an evaluative measure of cognitive function [42].

### Enzyme-Linked Immunosorbent Assay

Following the manufacturer’s protocol, the NGF concentration in each brain sample was measured using a Mouse Beta-NGF Enzyme-linked Immunosorbent Assay (ELISA) Kit (NBP2-81189, Novus). The colorimetric reaction product was measured at 450 nm using a microplate reader (Thermo Scientific Spectrophotometer VarioSkan LUX, Singapore). Each test was performed in duplicate, and the data were expressed as picograms per milligram (pg/mg) protein and expressed as mean ± SEM. After MCAO, the rats were treated once, and the experimental method was the same.

### TUNEL Staining

The survival of neurons in the hippocampus after MCAO/R was evaluated using TUNEL staining. The numbers of TUNEL-positive and DAPI-positive cells in the right hippocampal CA1, CA2, CA3, and DG regions were counted using TUNEL staining. The details of the experiment are provided in Appendix S1.

### Immunohistochemistry

Immunohistochemistry quantized Ach through choline acetyltransferase (ChAT), vascular endothelial growth factor A (VEGFA), and p-NF-κB p65. Specific experimental details can be found in Appendix S1.

### Western Blot

Total protein was obtained from the hippocampus of infarcted rats. The same amounts of protein samples were separated by 8% SDS-PAGE gel and then transferred to the PVDF membrane. These membranes were then blocked with 5% skimmed milk powder before being probed with the following primary antibodies: VEGFA (1:1000, 19003–1-AP, Proteintech); p-p65 (1:1000, sc-166748, Santa Cruz); nuclear factor-κB (NF-κB) NF-κB p65 (1:1000, D14E12, Cell Signaling Technology); actin (1:5000, EM21002, HUABIO); occludin (1:1000, 71–1500, Invitrogen); ZO-1 (1:1000, 61–7300, Invitrogen). On the second day, horseradish peroxidase-labeled goat anti-rabbit antibody (1:10000, Jackson, 111–035-003) and horseradish peroxidase-labeled goat anti-mouse antibody (1:10000, Jackson, 115–035-003) was added. After incubation for 1 h, an ultra-hypersensitive ECL chemiluminescence kit (Beyotime, China) was used for visualization using an ImageQuant LAS 4000 (GE, USA). Western blotting (WB) data were analyzed using ImageJ software. Specific experimental details can be found in Appendix S1.

### Grouping

**Fig. 1:**
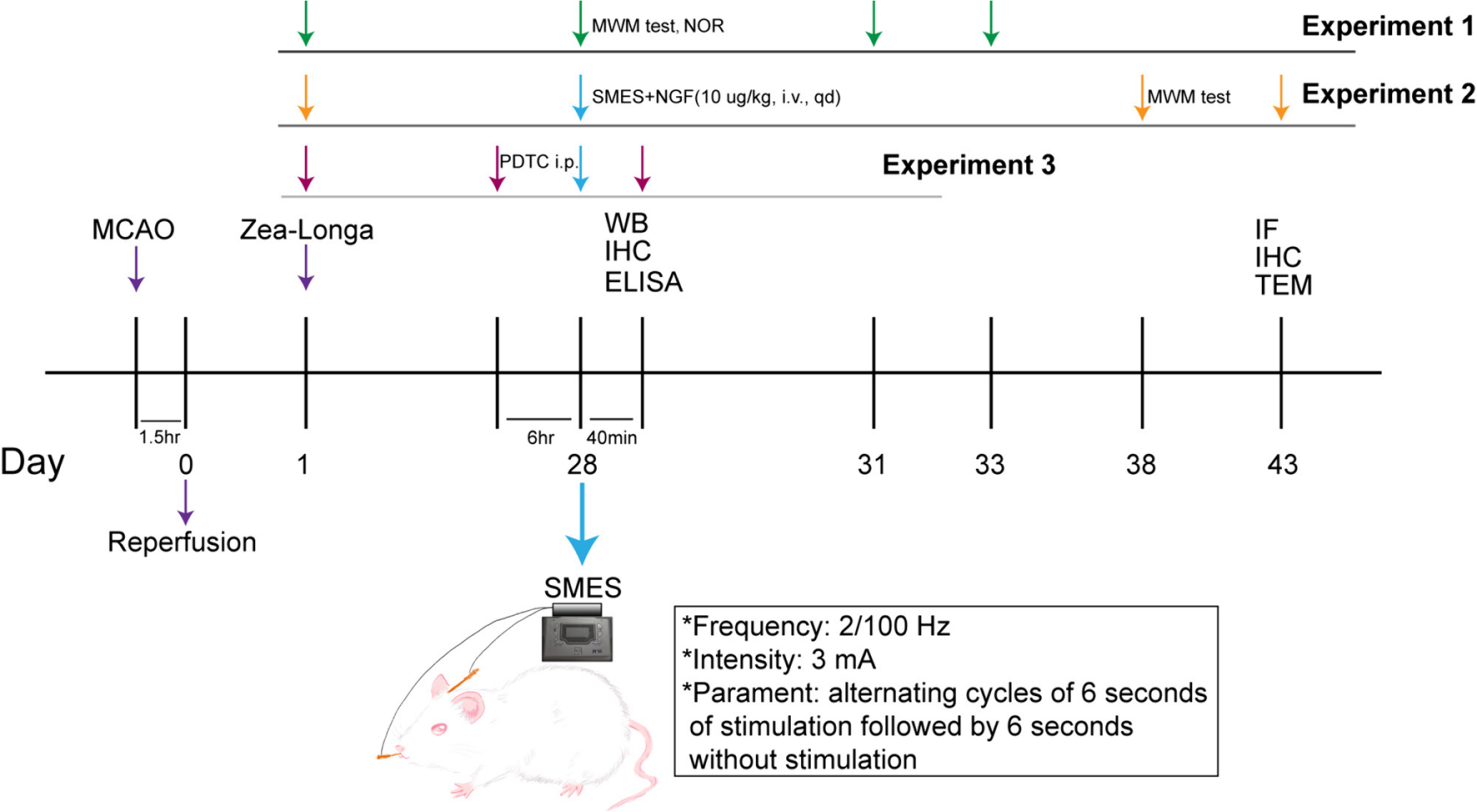
Experimental framework. Experiment 1: The study divided rats into control and MCAO/R groups to test memory impairment after modeling. The control group received no intervention, while the MCAO/R group was modeled using the suture method and tested with water maze or object recognition experiments 28 days later. Experiment 2: The study tested the continuous therapeutic effect by dividing MCAO/R rats into different groups and using normal rats as a control. After treatment, a water maze experiment was conducted for 6 days, followed by detection of IF, IHC, and TEM. Experiment 3: MCAO/R rats were divided into different groups for ELISA testing to verify the permeability of mNGF in various brain regions by SMES. Another set of MCAO/R rats were divided into groups for WB and ICH verification of the p65-VEGFA-TJs signal axis to explore the effect of SMES on opening the BBB of the hippocampus

Figure 1 shows the grouping of the three experiments.

#### Experiment 1

 In the study investigating potential memory impairment following modeling, rats were stratified into a control group and MCAO/R group. Rats in the control group underwent no interventions. Memory assessments were conducted using the MWM or NOR experiment 28 days post-modeling.

#### Experiment 2

In the study aimed at confirming the sustained therapeutic efficacy, MCAO/R rats were randomly allocated into four groups: MCAO/R group, NGF group, SMES group, and NGF + SMES group, with normal rats were served as the control group. Following the intervention, a 6-day water maze task was conducted, followed by the assessment of IF, IHC, and TEM analyses.

#### Experiment 3

In order to assess the permeability of mNGF in various brain regions of MCAO/R rats by SMES, MCAO/R rats were randomly assigned to different groups including MCAO/R group, NGF group, NGF + PDTC + SMES group, and NGF + SMES group for ELISA, to investigate the impact of the p65-VEGFA-TJs signaling pathway on SMES-induced opening of the BBB in the hippocampus of MCAO/R rats to facilitate mNGF entry into the brain. Therefore, the MCAO/R rats were randomly divided into the MCAO/R group, PDTC + SMES group, and SMES group for WB and ICH verification of signaling pathway proteins.

### Quantification and Statistical Analysis

GraphPad Prism 8.0 (Graph Pad, USA) was used for statistical analysis. All data were expressed as mean ± standard error of the mean (SEM). Only data with normal distribution could be calculated. In order to establish a difference in two groups, an independent sampling test (*t*-test) was used. Ordinary one-way ANOVA and two-way repeated measure ANOVA were used to analyze differences between multiple groups. The value of *P* < 0.05 was considered statistically significant (see Supplementary Table 1 for detailed statistical reports) [40].

## Result

### The Through of mNGF into Different Cognitive Brain Regions by SMES

**Fig. 2:**
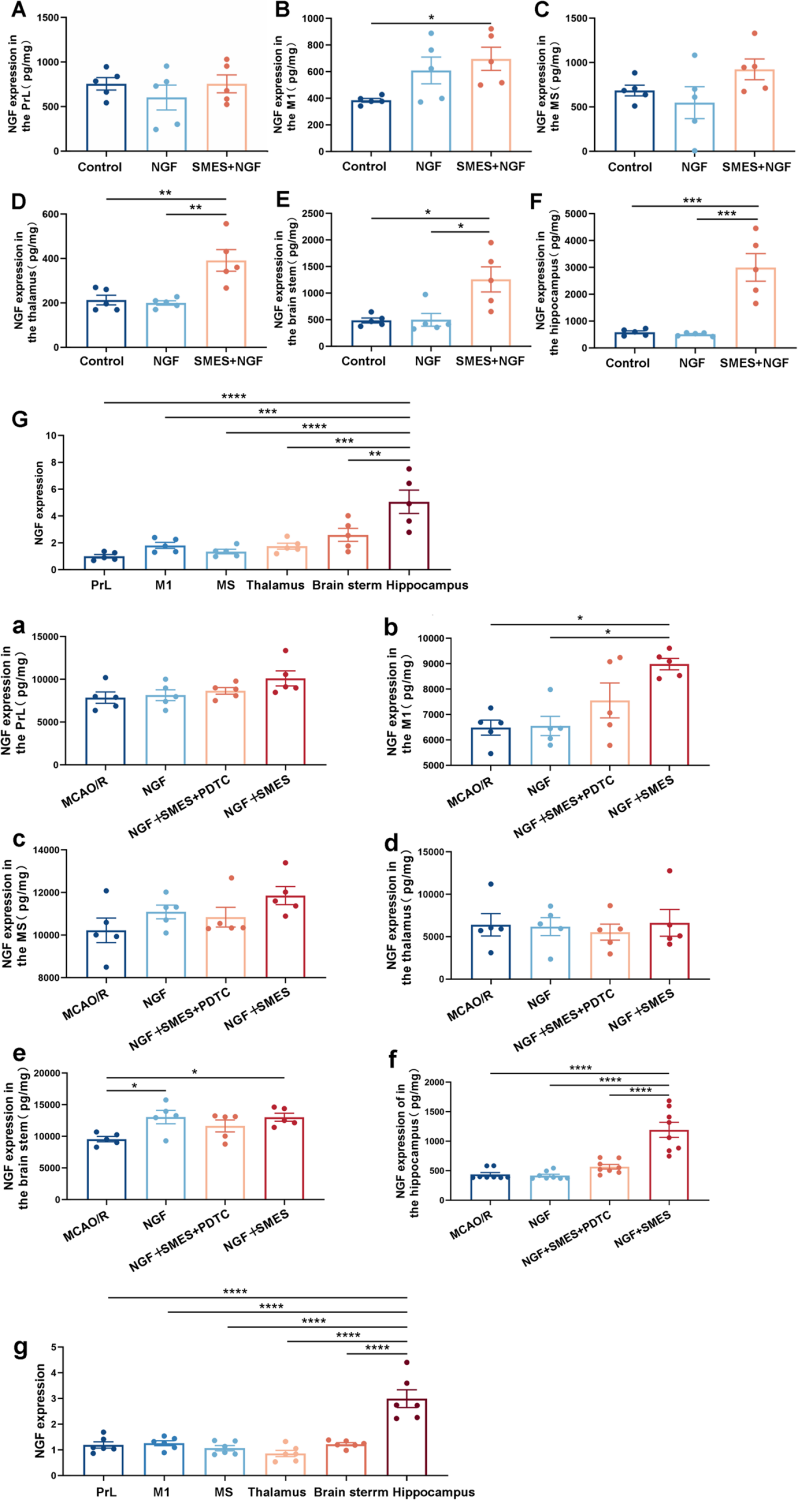
The protein of mNGF expression in the M1 and various cognitive-related brain regions as measured by ELISA. **A**, **B**, **C**, **D**, **E**, **F** The NGF + SMES group had significant effect except PrL and MS. The results are demonstrated as the mean ± SEM (*n* = 5, each group).^*^P < 0.05, ^**^P < 0.01, ^***^P < 0.001, and ^****^P < 0.0001 vs. NGF + SMES group. **G** The hippocampus had significant effect. The results are demonstrated as the mean ± SEM (*n* = 5, each group). ^**^P < 0.01, ^***^P < 0.001, and ^****^P < 0.0001 vs. hippocampus group. **a**, **b**, **c**, **d**, **e**, **f** Compared with the MCAO/R group, NGF group, or NGF + SMES + PDTC group, NGF + SMES group had significant permeation effect in the M1, brain stem, and hippocampus. The results are demonstrated as the mean ± SEM (*n* = 5 or 8, each group). ^*^P < 0.05, ^**^P < 0.01, and ^****^P < 0.0001 vs. NGF + SMES group. **g** The hippocampus had significant effect. The results are demonstrated as the mean ± SEM (*n* = 6, each group). ^****^P < 0.0001 vs. hippocampus group

In our previous experiments, we observed that SMES has the potential to enhance mNGF penetration into the frontal lobe of rats [33]. However, the extent of mNGF penetration into different cognitive-related brain regions remains unknown. We measured and compared mNGF permeability and specific permeability in cognitive-related brain regions of the three groups of normal rats to validate these effects in rats. Thus, SMES facilitated the upregulation of mNGF in the primary motor cortex (M1) and various cognitive-related brain regions, except for the prefrontal lobe (PrL) (Fig. 2A–F). A significant increase in mNGF levels was observed in the hippocampus (Fig. 2G).

Given the promising performance of the SMES in the hippocampus, we used ELISA to assess the permeability of mNGF in the affected hemispheres of the three groups of MCAO/R rats. As anticipated, mNGF levels were elevated in the NGF + SMES group 28 d after MCAO/R (Fig. 2a, b, c, d, e, f). These findings suggest that SMES may accelerate the transmission of mNGF across the BBB, particularly to the hippocampus.

### Spatial Learning and Memory Were Still Affected 28 Days After MCAO/R

**Fig. 3:**
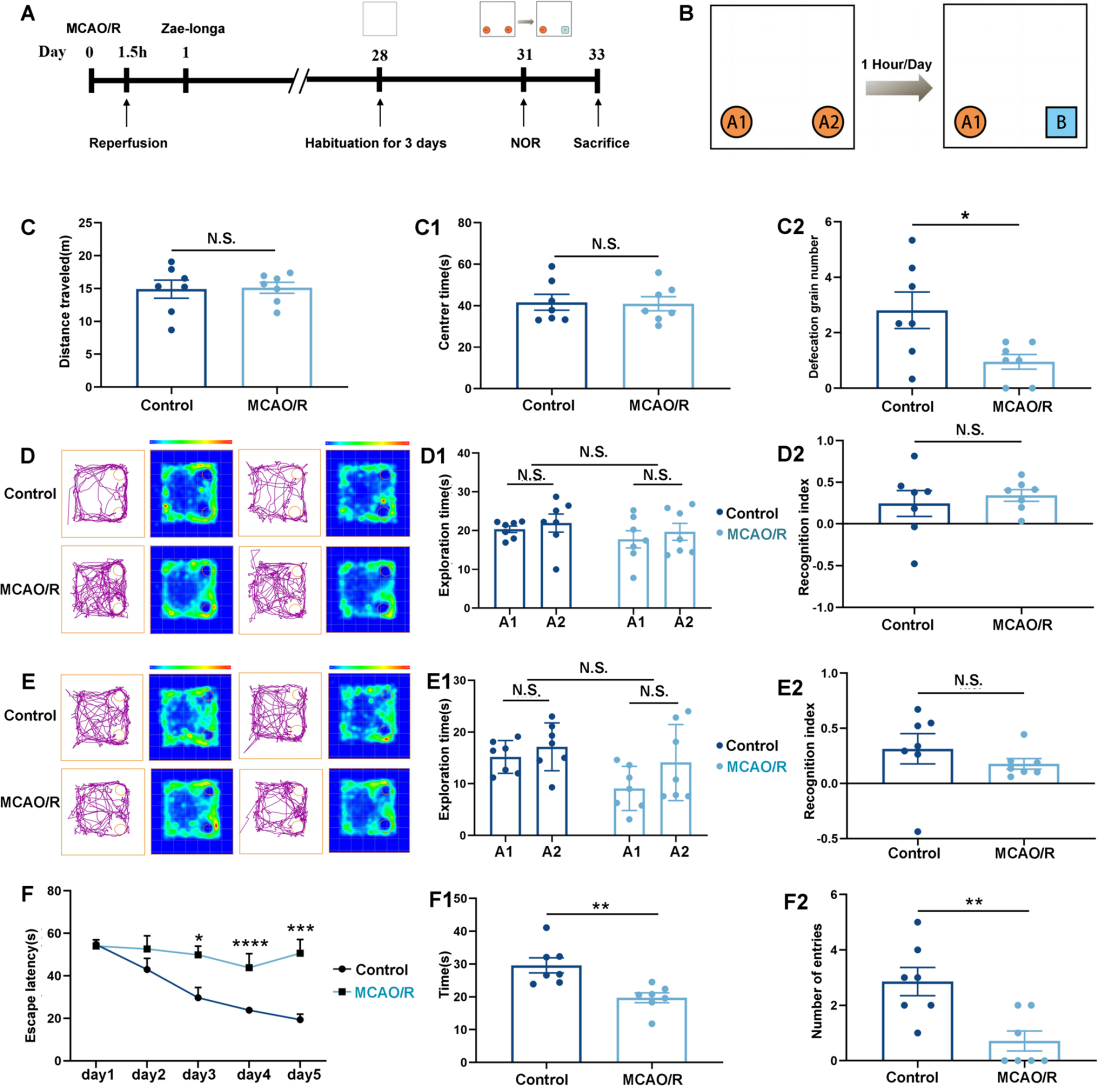
MCAO/R modeling for 28 days still resulted in impaired spatial learning ability and long-term memory in rats. **A** The new object recognition experiment (NOR) test intervention diagram. **B** During the training period, A1 and A2 objects were placed in the box, and A2 was replaced with B object after 1 h or 24 h. **C**, **C1**, **C2** The distance traveled, the center time, and the number of defecation grains during the habituation period. **D**, **D1**, **D2** and **E**, **E1**, **E2** The typical traces and heat map from the NOR test results; exploration time with A1 and A2 and the recognition index represented the recognition memory in two groups. **F**, **F1**, **F2** Escape latency in the Morris water maze (MWM) test for 5 training days in two groups. The time spent and number of entries in the target quadrant during the sixth day (probe) in the MWM test. The results are demonstrated as the mean ± SEM (*n* = 7, each group). ^**^P < 0.01, ^***^P < 0.001, and ^****^P < 0.0001 comparison with the first day

We conducted a NOR test and an MWM test to evaluate the cognitive function of rats after MCAO/R28 days (Fig. 3A, B) [38, 41]. There was no significant change in spatial cognitive ability in the 28 rats after MCAO/R, and there were no obvious abnormalities in excitability (Fig. 3C). The central grid residence time in the open field analysis reflected spatial cognitive ability, and there was no significant difference between the groups (Fig. 3C1). The number of fecal particles in the open-field analysis of animals reflects the degree of animal tension (Fig. 3C2). The greater the number of fecal particles, the higher the tension. The number of fecal particles in the control group was significantly higher than in the MCAO/R group. Tension in the rats decreased 28 days after MCAO/R. In the short-term memory and long-term memory tests of NOR, the main effect of group, time, group, and time/group interaction was not significant (Fig. 3D–E2). The NOR experiment showed that the long-term memory and short-term memory of the two groups of rats were not significantly different because new object recognition was attributed to frontal lobe damage, which indirectly proved that the frontal lobe of MCAO/R 28 rats was not damaged (Fig. 3D–E2). Next, we performed the water maze test. In the water maze test before treatment, the main effect of group, time, group, and time/group interaction all had significant effects (Fig. 3F). The control group showed strong learning ability in the 5-day experiment, and the escape latency was gradually shortened. Although the escape latency of the MCAO/R group on the third and fourth days was improved compared to that on the first day, the escape latency on the last day was longer (Fig. 3F). After the platform was removed on the sixth day, the rats in the control group showed a superior learning bias when navigating to the target quadrant. Compared with the MCAO/R group, they showed more target quadrant residence time and an increased frequency of platform crossover in the target quadrant (Fig. 3F1, F2). Our results showed that the spatial learning and memory of rats 28 days after MCAO/R were still damaged because MWM is the most sensitive detection index of the hippocampus and the significance of SMES opening the hippocampal BBB. Therefore, in the next experiment, we focused on the improvement of the hippocampus by treatment [43].

### mNGF Mediated by SMES Improved Spatial Learning and Memory

**Fig. 4:**
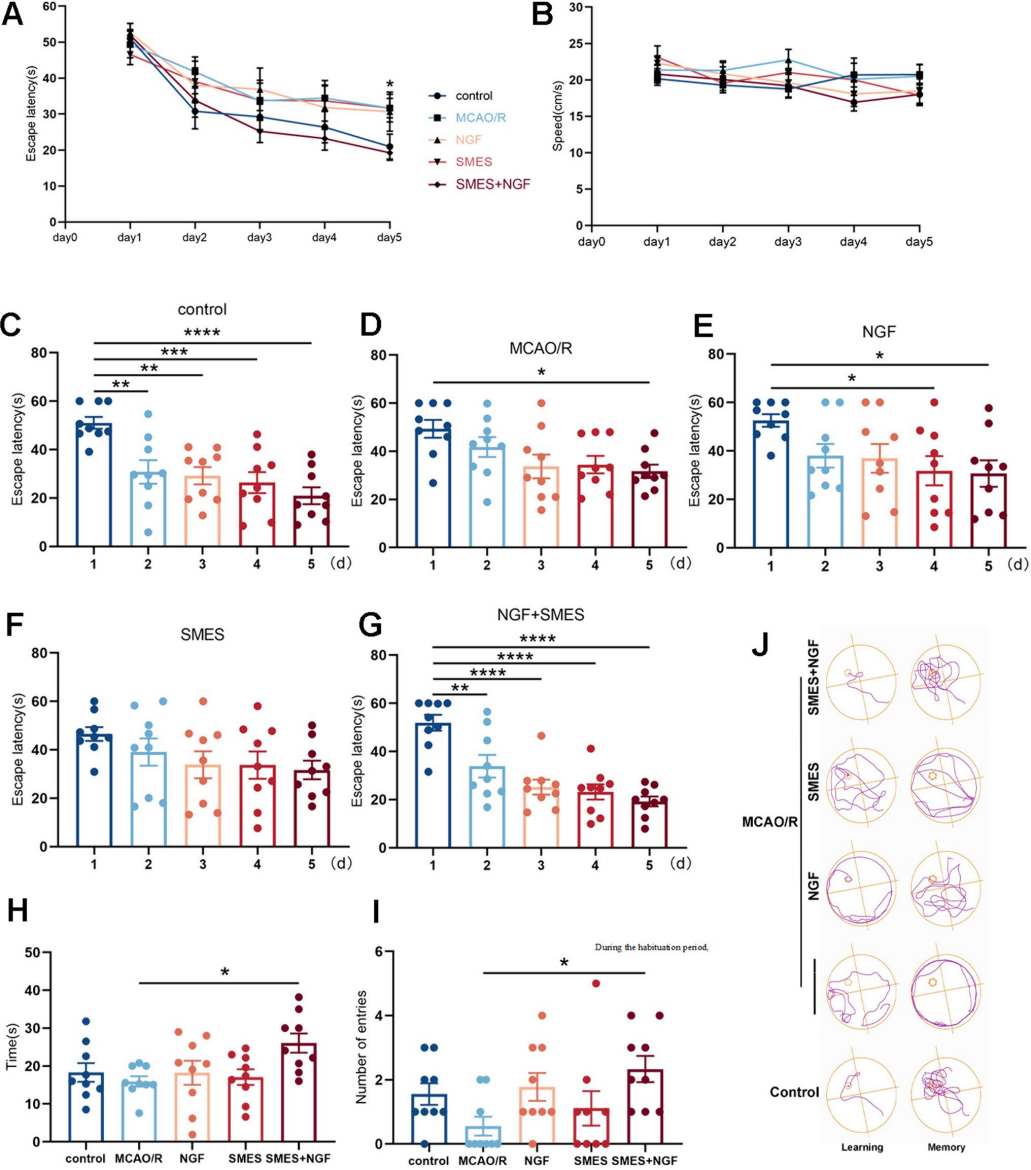
Specific mode electroacupuncture stimulation delivered mNGF into the brain improved spatial learning ability and long-term memory in rats. **A** Escape latency and **B** swimming speed (**C**, **D**, **E**, **F**, **G**) in MWM test for 5 training days in different groups. The results are demonstrated as the mean ± SEM (*n* = 9, each group). ^*^P < 0.05, ^**^P < 0.01, ^***^P < 0.001, and ^****^P < 0.0001, comparison with the first day. **H** The time spent in the target quadrant and **I** number of platform crossings during the sixth day (probe) in the MWM test. **J** The swimming paths of rats are observed following modeling, and representative image is captured for the NGF + SMES group during the water maze test on day 5 (learning) and day 6 (memory). The platform removed on the sixth day is represented by a dotted line. The results are demonstrated as the mean ± SEM (*n* = 9, each group).^*^P < 0.05 vs. MCAO/R group

This study aimed to examine the influence of spatial learning and memory on a short-term memory task dependent on hippocampal processing. Before the test, we carried out continuous treatment (Fig. 1 Experiment 2). Repeated measure two-way ANOVA analysis demonstrated that the learning curves of the groups were different with a main effect of the day factor (Fig. 4A). The findings revealed that rats in the SMES + NGF group exhibited decreased escape latency and path length when the concealed platform was located during the training session, suggesting an enhancement in their learning ability following continuous SMES + NGF treatment. In contrast, the rats in the other groups did not display any notable reduction in latency or path length (Fig. 4A, C–G). There were no notable variations in swimming speed across any of the experimental groups (Fig. 4B). Notably, on the sixth day, the rats in the NGF + SMES group exhibited a superior learning bias in their navigation towards the target quadrant. They demonstrated reduced time and travel distance and increased frequency of platform crossings within the goal quadrant compared to the MCAO/R group (Fig. 4H–J).

### mNGF Mediated by SMES Reduced Hippocampal Cholinergic Neuron Apoptosis and Mitophagy

**Fig. 5:**
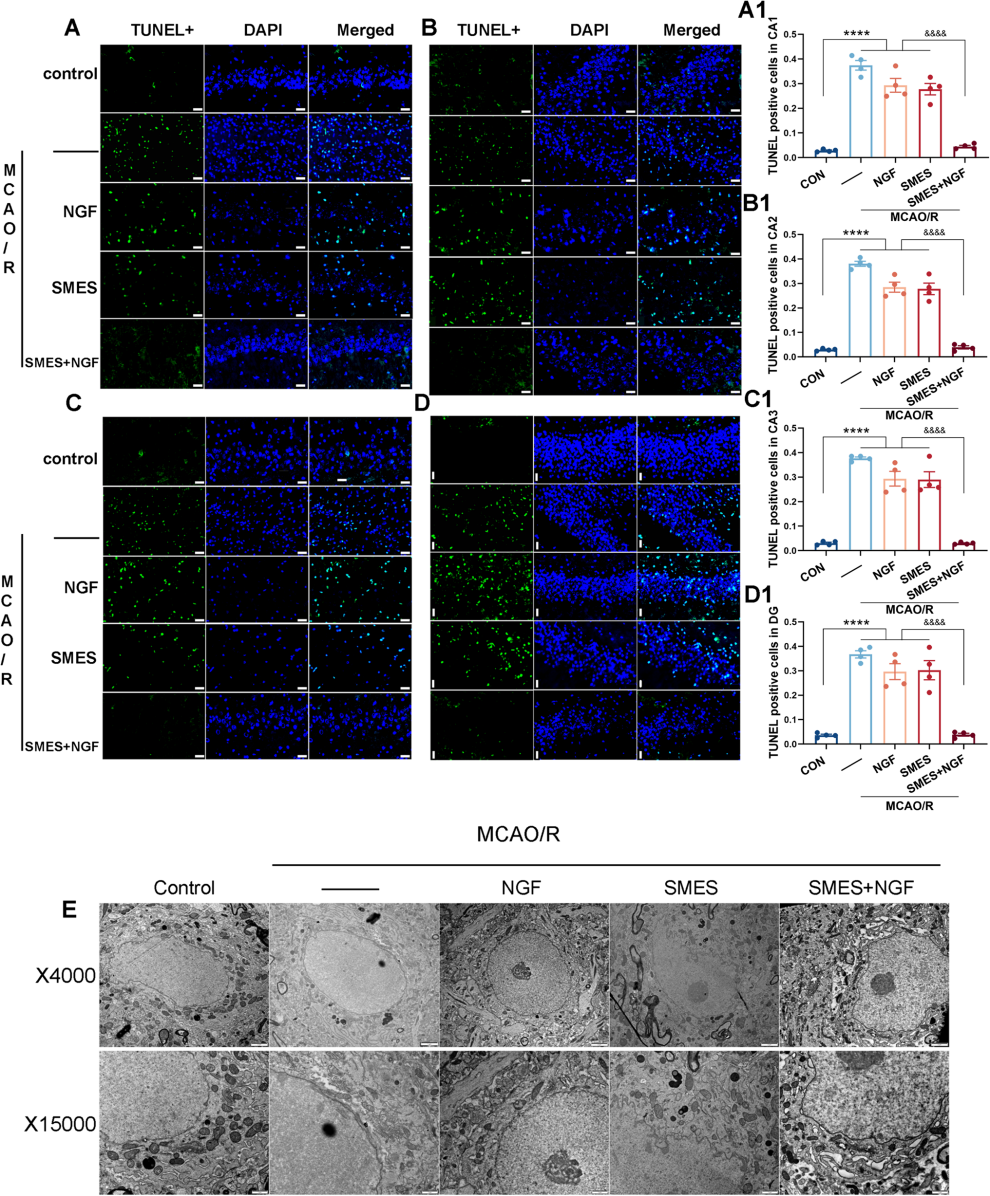
The expression of TUNEL cells in different parts of the hippocampus and the mitochondrial state in the CA1 area after treatment in each group. **A**, **B**, **C**, **D** TUNEL-stained apoptotic cells remained present in various partitions of the hippocampus 28 days after modeling, but the SMES + NGF group was less expressed; the scale bar is 20 µm. **A1**, **B1**, **C1**, **D1** Immunohistochemical analysis number of TUNEL-positive cells divided by DAPI staining cell ratio in the hippocampus after MCAO/R in rats. **E** The mitochondria surrounding neurons in the MCAO/R and NGF groups exhibited impairments, characterized by abnormalities in crest structure and the presence of vacuoles. Conversely, the mitochondria in the remaining groups did not demonstrate significant damage. Mean ± SEM (*n* = 4). The significant levels: ^****^P < 0.0001 vs. control group, ^&&&&^*P* < 0.0001 vs. SMES + NGF group

Binding of SMES and NGF had been observed to reduce hippocampal apoptosis and mitophagy following MCAO/R. TUNEL-positive cells were detected in various hippocampus regions within each group, with a lower incidence of apoptosis observed in both the control and SMES + NGF groups. Conversely, the remaining groups exhibited more apoptotic cells than the control and SMES + NGF groups, indicating that the combination of SMES and NGF effectively mitigated apoptosis within the hippocampus (Fig. 5A–D1).

Examination of mitochondria in the CA1 region of the hippocampus revealed normal mitochondria, a complete double membrane, and a clear crest in the control, SMES, and SMES + NGF groups. However, most mitochondria in the MCAO/R and NGF groups displayed abnormal crest structures, with many swollen mitochondria containing numerous small vesicular crests that were not connected to the outer chambers. Consequently, this resulted in structural disturbances in the mitochondrial crest, the appearance of blank areas, and degeneration of the outer mitochondrial membranes (Fig. 5E).

### SMES and NGF Increased the Number of Cholinergic Neurons in the Infarcted *Hippocampus*

The expression levels of ChAT-positive cells in the four parts of the hippocampus were determined after MCAO/R to explore further the changes in the protein expression of ChAT-positive cells in each group. Immunohistochemical analysis of ChAT was performed to examine whether MCAO/R and its treatment regulated protein expression in cholinergic neurons.

**Fig. 6:**
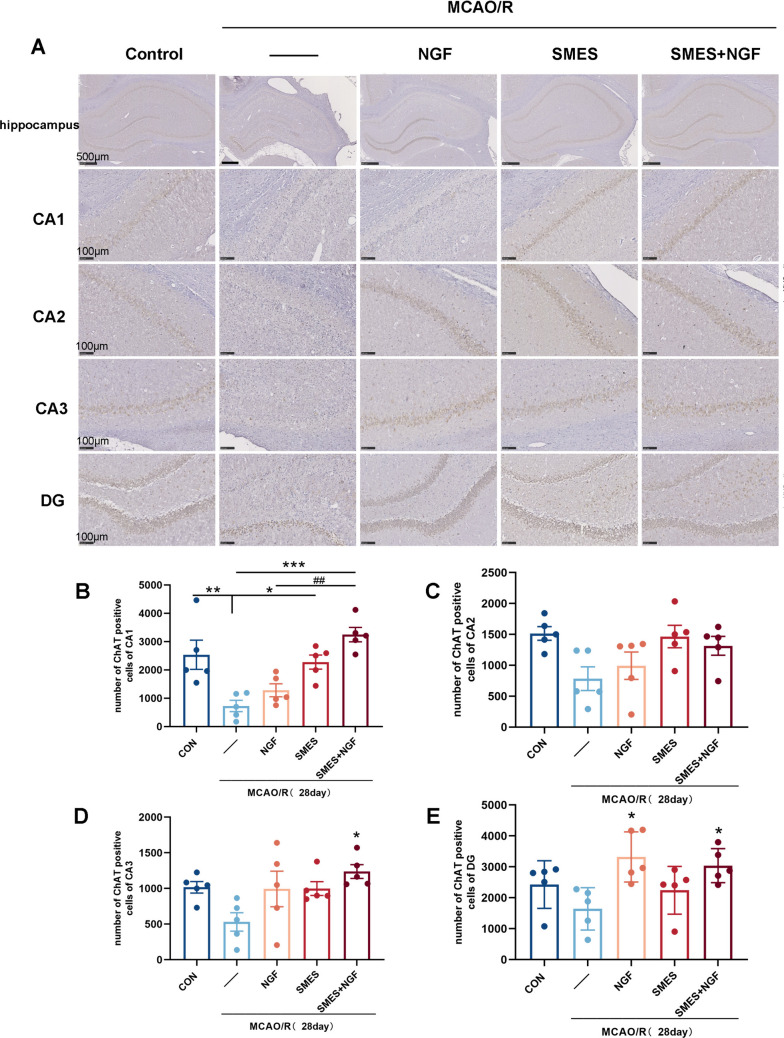
The expressions of the number of ChAT-positive cells at different parts of the hippocampus after treatment in rats. **A** Representative immunohistochemical images of ChAT-positive cells in the hippocampus and its various partitions after MCAO/R in rats. **B**, **C**, **D**, **E** Immunohistochemical analysis number of ChAT-positive cells in the hippocampus of ischemic parietal cortex after MCAO/R in rats. Mean ± SEM (*n* = 5). The significant levels: ^*^P < 0.05, ^**^P < 0.01, and ^***^P < 0.001 vs. MCAO/R group and ^##^*P* < 0.01 vs. NGF group

As shown in Fig. 6A, immunohistochemical analysis revealed that the protein expression of ChAT-positive cells in the hippocampal CA1 region in the SMES + NGF group was significantly higher than that in the MCAO/R, NGF, and SMES groups (*P* < 0.01; Fig. 6B). In the hippocampal CA2 region, there were no significant differences between the groups (Fig. 6C). In the hippocampal CA3 region, the SMES + NGF group, compared with the MCAO/R group, the number of ChAT-positive cells increased significantly (*P* < 0.05; Fig. 6D). In the hippocampal DG region, the SME + NGF and NGF groups, compared with the MCAO/R group, the number of ChAT-positive cells increased significantly (*P* < 0.05; Fig. 6E).

Cholinergic neurons in the hippocampus of the control and SMES + NGF groups were arranged regularly with clear layers. Neurons in the MCAO/R, NGF, and SMES groups were arranged irregularly, with larger intercellular spaces than those in the normal and SMES + NGF groups, and a few neurons lost structural integrity.

### SMES Can Promote the p65-VEGFA-TJs Signaling Pathway Activation

Research has demonstrated that VEGFA induces activation of brain microvascular endothelial cells, while the well-established NF-κB/MMP-9 axis can elicit VEGF release [44–46]. These discoveries imply that the activation of the NF-κB signaling pathway fosters angiogenesis and neurogenesis, thereby warranting additional investigation into the downregulation of VEGFA on tight junctions.

**Fig. 7:**
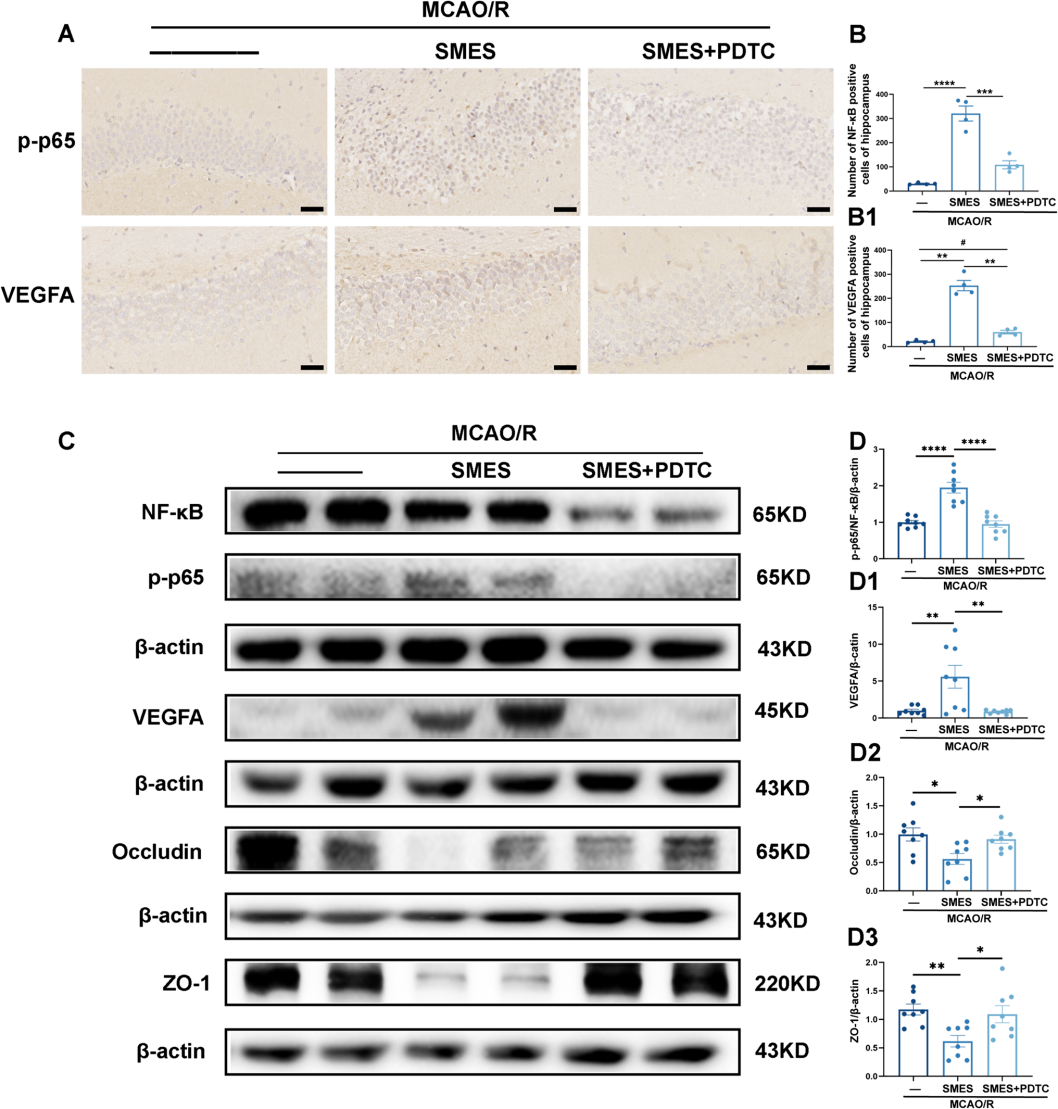
SMES altered the expression of p-p65, VEGFA, and TJs in the hippocampus. **A** Number of VEGFA-positive cells and NF-κB nuclear staining was visualized in brown color. Scale bar, 50 µm. **B**, **B1** Quantification of the immunohistochemistry results; *n* = 4 in each group. **C** Western blot analysis of NF-κB, p-p65, VEGFA, occludin, and ZO-1 expression in infarcted side hippocampus from each group. **D**, **D1**, **D2**, **D3** Quantification of the Western blot results, left panel shows summarized protein expression normalized to β‐actin; *n* = 8 in each group. ^*^P < 0.05, ^**^P < 0.01, ^***^P < 0.001, and ^****^P < 0.0001 vs. SMES group and ^#^*P* < 0.05 vs. MCAO/R group, one-way ANOVA followed by Bonferroni post hoc test

We conducted the experiment of the inhibitor group (Fig. 1 Experiment 3). We observed that SMES treatment resulted in the activation of hippocampal p-p65, leading to downstream modulation of the VEGFA protein and a subsequent reduction in TJ expression in the BBB. Immunohistochemistry was employed to validate the phosphorylated NF-κB and VEGFA expression. A comparative analysis was conducted, including multiple groups. The findings depicted in Fig. 7A, B, and B1 demonstrate that the SMES group exhibited a significantly higher number of positive cells post-treatment compared to the MCAO/R group and the SMES + PDTC group, suggesting that SMES had the potential to enhance nuclear NF-κB expression and cytoplasmic VEGFA expression.

The levels of NF-κB, p-p65, VEGFA, occludin, and ZO-1 were assessed through WB analysis in the hippocampus of the infarcted side within each experimental group. These findings revealed that SMES induced the activation of phosphorylated p65 and increased VEGF-A expression, reducing the expression of occludin and ZO-1, which are crucial components of tight junction proteins (Fig. 7C–D3).

## Discussion

Ischemic stroke is a prominent etiological factor for cognitive impairment on a global scale, primarily attributed to the apoptosis of a substantial population of cholinergic neurons [1, 2]. Regrettably, numerous neuroprotective agents have proven ineffective owing to their inherent biological toxicity, severe adverse reactions, and limited efficacy [47, 48]. Within this investigation, alterations in hippocampal neurons in the affected hemisphere were still evident even after 28 days following MCAO/R. This study revealed a decline in the number of cholinergic neurons within the hippocampus on the affected side, accompanied by an increase in mitophagy and apoptosis, ultimately leading to impaired spatial learning and memory in rats. Our treatment has the potential to alleviate these conditions. Specifically, SMES promotes the activation of the p65-VEGFA-TJs pathway, thereby significantly increasing the level of exogenous NGF in the hippocampus of MCAO/R rats. Introduction of NGF into the brain enhanced hippocampal synaptic plasticity in MCAO/R rats. Furthermore, SMES effectively opened the BBB without detrimental effects on hippocampal cholinergic neurons, mitochondrial integrity, or autophagy.

**Fig. 8:**
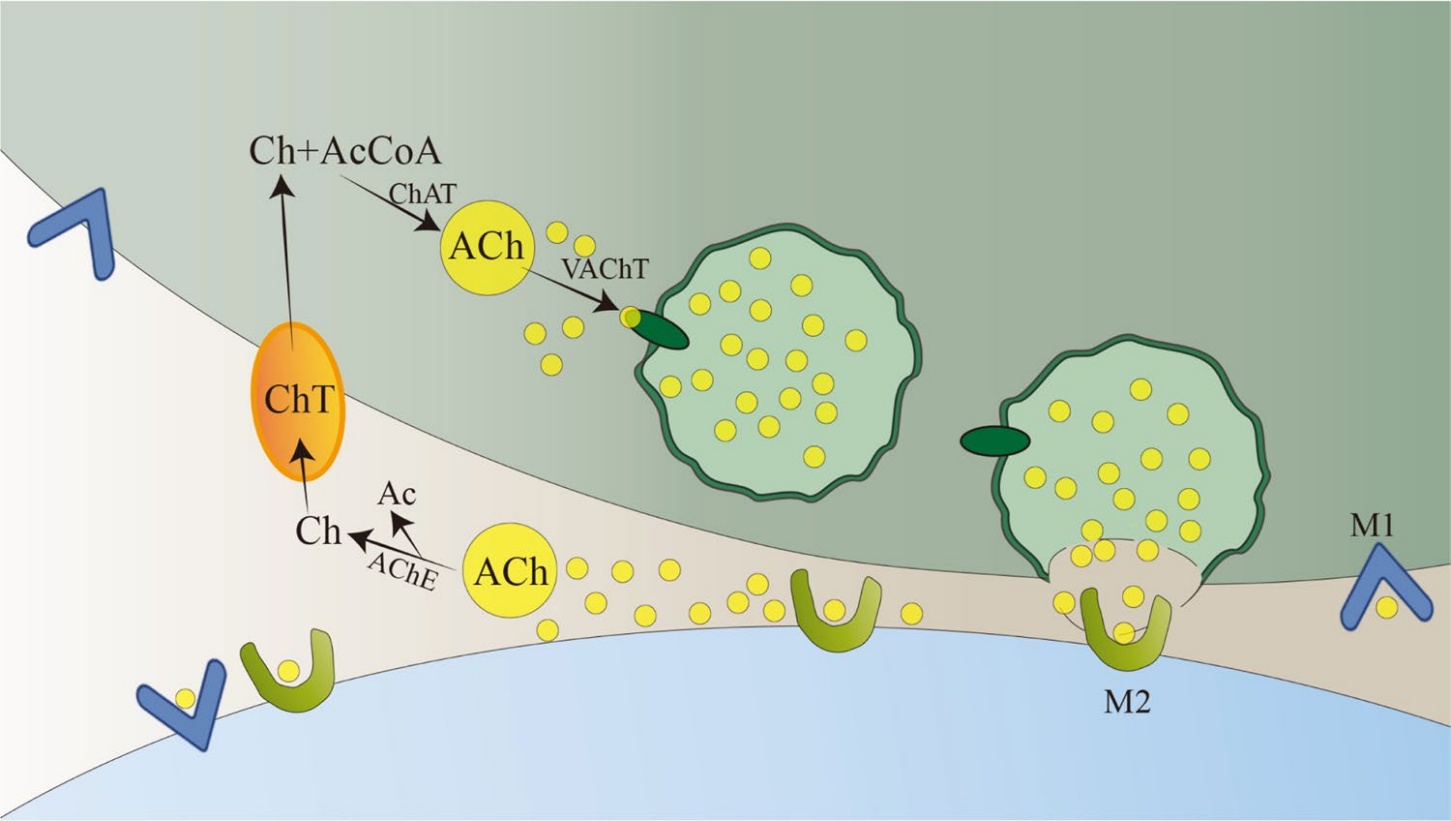
The key steps in the synthesis, release, and reuptake of neurotransmitter acetylcholine are illustrated

The central cholinergic system is closely associated with learning and memory. Acetylcholine (Ach) is an important neurotransmitter in the central cholinergic system, including acetylcholinesterase (AChE) and butyrylcholinesterase (BChE) [49, 50]. Choline (Ch) and acetate synthesize ChAT, which is stored in vesicles under the action of the vesicular acetylcholine transporter (VAChT) (Fig. 8) [51, 52]. Its main function is maintaining consciousness and playing an important role in attention, learning, and memory [53]. It is a major neurotransmitter in the brain. BFCN is the main target cell of NGF after entering the brain [54]. NGF can promote the growth, repair, and survival of BFCN [55]. NGF can reduce the damage to the cholinergic system and the impact after injury. In the normal brain, these neurotrophic factors are produced by the cortical target cells of the basal forebrain cholinergic projections and locally in the cholinergic cell body region. Therefore, the availability of neurotrophic factors, whether in the axon terminal region or the cell body region, may contribute to the development and maintenance of the cholinergic system and may also contribute to its recovery after injury [18].

Our previous study found that SMES acts on Shuigou and Baihui can open the BBB in normal rats. SMES-promoted BBB opening did not lead to brain edema, glial cell activation, or apoptosis. It was also confirmed that electroacupuncture could induce BBB opening in rats by increasing cerebral blood flow and activating cortical neurons in the brain tissue through SMES [26, 27, 34].

Research has demonstrated that the phosphorylation of NF-κB in brain microvascular endothelial cells led to a decrease in the expression of tight junction proteins occludin and ZO-1 [44, 46]. Additionally, studies have revealed that the activation of VEGFA on brain microvascular endothelial cells could mitigate cerebral ischemia/reperfusion injury [45]. Furthermore, the classical NF-κB/MMP-9 axis could stimulate the release of VEGF [56, 57]. These findings suggested that the activation of the NF-κB signaling pathway promoted angiogenesis and neurogenesis, and the downregulation of VEGFA on tight junctions warrants further investigation. Our experimental findings demonstrated that activation of NF-κB phosphorylation in brain microvascular endothelial cells during repairing cerebral ischemia led to upregulating VEGFA expression. Consequently, this reduces the expression of tight junction proteins, resulting in the opening of the hippocampal BBB and facilitating the entry of NGF into the brain.

In future investigations, it will be imperative to delve deeper into the effects of SMES on cerebral ischemic injury, encompassing not only nerve regeneration but also neural development, axonal plasticity, and cerebrovascular repair.

## Conclusion

Recent research has demonstrated that ischemic stroke can reduce the population of cholinergic neurons within the rat hippocampus during the recuperation phase and affect spatial learning and memory. SMES facilitates the upregulation of mNGF in M1 and various cognition-related brain regions, except for the PrL. By activating the p65-VEGFA-TJs pathway, SMES effectively opened the BBB in rats and facilitated the transportation of NGF into the hippocampus. Following administration, SMES exhibited enhanced spatial learning and memory in rats while concurrently augmenting the number of cholinergic neurons within the hippocampus. This discovery provides innovative evidence supporting the potential of macromolecular therapeutic drugs for treating central nervous system disorders.

## Supplementary Information

Below is the link to the electronic supplementary material.

Supplementary file1 (DOCX 71245 KB)

Supplementary file2 (DOCX 27 KB)

## Data Availability

The data that support the findings of this study are available from the corresponding author upon reasonable request.

## Abbreviations

PSCI: Post-stroke cognitive impairment
NGF: Nerve growth factor
BFCN: Basal forebrain cholinergic neurons
BBB: Blood-brain barrier
SMES: Specific mode electroacupuncture stimulation
CCA: Common carotid artery
ECA: External carotid artery
ICA: Internal carotid artery
mNGF: Mouse nerve growth factor
PDTC: Pyrrolidine dithiocarbamate
Shuigou: GV26
Baihui: GV20
TEM: Transmission electron microscopy
MWM: Morris water maze
NOR: Novel object exploration test
ChAT: Choline acetyltransferase
ELISA: Enzyme-linked immunosorbent assay
VEGFA: Vascular endothelial growth factor A
WB: Western blotting
M1: Primary motor cortex
PrL: Prefrontal lobe
Ach: Acetylcholine
AChE: Acetylcholinesterase
BChE: Butyrylcholinesterase
Ch: Choline
VAChT: Vesicular acetylcholine transporter
ChT: Choline transporter
