# Supplementary material for: Specific Mode Electroacupuncture Stimulation Mediates the Delivery of NGF Across the Hippocampus Blood–Brain Barrier Through p65-VEGFA-TJs to Improve the Cognitive Function of MCAO/R Convalescent Rats
Source: Mol Neurobiol. 2024 Jul 12;62(2):1451–66. doi: 10.1007/s12035-024-04337-8 (PMC11772513; doi:10.1007/s12035-024-04337-8)
Supplement: Supplementary file 1 — Supplementary file1 (DOCX 71245 KB) [file 12035_2024_4337_MOESM1_ESM.docx]

**Method Details：**

**1.Immunofluorescence and TUNEL staining**

MCAO/R rats were subjected to TUNEL staining after 10 days of treatment. The rats were deeply anesthetized and infused with 0.9% normal saline through the heart, followed by 4% paraformaldehyde（PFA; Tianjin Institute of Chemical Reagents, 20051208）/ PBS solution. The whole brain was taken and fixed in 4%PFA, followed by 15% and 30% sucrose aqueous solution, and dehydrated at 4 °C gradient. Then the brain tissue was sliced with a frozen section machine (-20°C，Thermo), and the slice thickness was 30μm.

TUNEL staining was performed according to the manufacturer 's instructions, and the sections were labeled with a fluorescein in situ cell death detection kit (in situ cell death detection kit, Roche Diagnostics Deutschland GmbH, 11684795910, Mannheim Germany). Then, the sections were stained with DAPI (Abcam, ab104139, American) for 5 min and sealed. The number of TUNEL, ChAT and DAPI positive cells in the CA1, CA3 and DG regions of the right hippocampus was counted. TUNEL positive cells were identified through immunofluorescence. Specifically, three microscope fields were chosen within the hippocampal CA1, CA2, CA3, and DG regions, and the green TUNEL positive cells were quantified. The TUNEL positive cell ratio was determined by averaging the number of TUNEL and DAPI double positive cells in three images per rat.

In order to calculate the permeability of FITC-NGF in different cognitive brain regions. During the data acquisition and processing phase, standardized protocols and procedures are employed to minimize errors. Specifically, three distinct areas measuring 1.5 mm^2^ each within the region were analyzed per section. The software ImageJ was utilized to identify the average fluorescence intensity, from which the mean value was computed. The mean, defined as the average fluorescence intensity (Mean = IntDen / Area), was subsequently documented.

**2. Western Blot**

  Grayscale analysis was employed to quantify the Western Blot bands, with darker colors corresponding to smaller gray values. Following calculation of the gray values for the target protein and internal reference protein (β-actin) on the same membrane using ImageJ software, normalization was conducted by dividing the gray value of the target protein by that of the internal reference protein in an Excel table, followed by statistical analysis.

**3.Immunohistochemistry**

After the intervention of MCAO/R rats in each group, the rats were anesthetized with 3% pentobarbital sodium injection, perfused with 0.9% normal saline through the heart, and then perfused with 4% paraformaldehyde. After dehydration, it was embedded in paraffin and sliced at a thickness of 4μm. After dewaxing, according to the manufacturer 's instructions, anticholine acetyltransferase antibody (HUABIO, ET1704-16,1:200), p-NFκB p65 antibody (Santa Cruz, sc-166748, 1:100), VEGFA antibody (Proteintech, 19003-1-AP, 1:200) were incubated overnight at 4°C by immunohistochemical staining, and then incubated with secondary antibodies (Abcam, goat anti-rabbit IgG,1:500 or Abcam, goat anti-mouse IgG,1:500). The sections were observed under an optical microscope.

The methodology employed in this study involved utilizing the positive staining cell counting method. Given that the positive reactions observed in this experiment were localized within the cytoplasm, the average count of positively stained cells within the cytoplasm was determined by examining random multiple microscope fields.

**Suppl. Fig. 1** The FITC-NGF assay was conducted to examine the exudation of FITC-NGF in brain amount of penetration, as indicated by the presence of green fluorescence staining. (A)In comparison to the control and FITC-NGF groups, the brain sections of FITC-NGF + SMES rats exhibited a notable intensity of green staining, suggesting that SMES enhanced BBB permeability. （B）In comparison to the control and FITC-NGF groups of MCAO/R rats, the brain sections of FITC-NGF + SMES rats exhibited a notable intensity of green staining, suggesting that SMES enhanced BBB permeability. （C）immunofluorescence analysis in different brain regions and the 4 regions of hippocampus of rats. Mean ± SEM (n=6). The significant levels: ^*^*P* < 0.05, ^**^*P* < 0.01， ^***^*P* < 0.001 and ^****^*P* < 0.0001 vs. FITC-NGF+SMES group and #P < 0.05 vs. FITC-NGF group. （D）immunofluorescence analysis in different brain regions and the 4 regions of hippocampus of MCAO/R rats. Mean ± SEM (n=5). The significant levels: ^****^*P* < 0.0001 vs. hippocampus and ^###^*P* < 0.001 and ^####^*P* < 0.0001 vs. The intervention of SMES did not result in any alteration in the permeability of FITC-NGF within the 4 regions of hippocampus of MCAO/R rats.
