# Supplementary material for: Specific Mode Electroacupuncture Stimulation Mediates the Delivery of NGF Across the Hippocampus Blood–Brain Barrier Through p65-VEGFA-TJs to Improve the Cognitive Function of MCAO/R Convalescent Rats
Source: Mol Neurobiol. 2024 Jul 12;62(2):1451–66. doi: 10.1007/s12035-024-04337-8 (PMC11772513; doi:10.1007/s12035-024-04337-8)
Supplement: Supplementary file 2 — Supplementary file2 (DOCX 27 KB) [file 12035_2024_4337_MOESM2_ESM.docx]

| **Fig.2** | **ELISA** | normal | PrL | ns | F(2,12)=0.6812, *P*=0.5245 | One-way ANOVA |
| --- | --- | --- | --- | --- | --- | --- |
|  |  |  | M1 | * | F(2,12)=4.338, *P*=**0.0382** |  |
|  |  |  | MS | ns | F(2,12)=2.172, *P*=0.1567 |  |
|  |  |  | thalamus | ** | F(2,12)=11.62, *P*=**0.0016** |  |
|  |  |  | brain stem | ** | F(2,12)=8.107, *P*=**0.0059** |  |
|  |  |  | Hippocampus | **** | F(2,12)=22.37, *P*<**0.0001** |  |
|  |  |  | Brain regions | **** | F(5,24)=11.39, *P*<**0.0001** |  |
|  |  | MCAO/R | PrL | ns | F(3,16)=2.252, *P*=0.1216 |  |
|  |  |  | M1 | ** | F(3,16)=7.231, *P*=**0.0028** |  |
|  |  |  | MS | ns | F(3,16)=2.186, *P*=0.1295 |  |
|  |  |  | thalamus | ns | F(3,16)=0.1424, *P*=0.9330 |  |
|  |  |  | brain stem | * | F(3,16)=4.187, *P*=**0.0229** |  |
|  |  |  | Hippocampus | **** | F(3,28)=27.75, *P*<**0.0001** |  |
|  |  |  | Brain regions | **** | F(5,30)=20.89, *P*<**0.0001** |  |
| **Fig.3** | **NOR** | Habituation | disstance | ns | *P*=0.4419 | T-test |
|  |  |  | traveled  center time | ns | *P*=0.4553 |  |
|  |  |  | defecation grain number | * | *P*=**0.0124** |  |
|  |  | Short-term memory | Interaction | ns | F(1,19)=0.06227, *P*=0.8056 | Repeated measure two-way ANOVA analysis |
|  |  |  | Day | ns | F(1,19)=1.870, *P*=0.1874 |  |
|  |  |  | Groups | ns | F(1,19)=1.879, *P*=0.0655 |  |
|  |  |  | Subject | ns | F(19,19)=1.829, *P*=0.0987 |  |
|  |  |  | Recognition index | ns | *P*=0.4665 | T-test |
|  |  | long-term memory | Interaction | ns | F(1,19)=0.05308, *P*=0.8203 | Repeated measure two-way ANOVA analysis |
|  |  |  | Day | ns | F(1,19)=2.120, *P*=0.1617 |  |
|  |  |  | Groups | ns | F(1,19)=1.841, *P*=0.1908 |  |
|  |  |  | Subject | ns | F(19,19)=1.484, *P*=0.1986 |  |
|  |  |  | Recognition index | ns | *P*=0.2944 | T-test |
|  | **MWM** | Escape latency(s) | Interaction | ** | F(4,96)=4.821, *P*=**0.0014** | Repeated measure two-way ANOVA analysis |
|  |  |  | Day | **** | F(2.861,68.68)=22.98, *P*<**0.0001** |  |
|  |  |  | Groups | ** | F(1,24)=13.68, *P*=**0.0011** |  |
|  |  |  | Subject | **** | F(24,96)=4.758, *P*<**0.0001** |  |
|  |  | Time(s) | | ** | *P*=**0.0015** | T-test |
|  |  | Number of entries | | ** | *P*=**0.0023** | T-test |
| **Fig.4** | **MWM** | Escape latency(s) | Interaction | ns | F(16,160)=0.7994, *P*=0.6847 | Repeated measure two-way ANOVA analysis |
|  |  |  | Day | **** | F(4,40)=11.23, *P*<**0.0001** |  |
|  |  |  | Groups | ns | F(4, 40) = 1.344, P=0.2705 |  |
|  |  |  | Subject | **** | F(40,160)=2.845, *P*<**0.0001** |  |
|  |  | Speed(cm/s) | Interaction | ns | F(16,180)=0.7337, *P*=0.7571 |  |
|  |  |  | Day | ns | F(4,45)=1.772, *P*=0.1511 |  |
|  |  |  | Groups | ns | F(3.441,154.9)=1.577, *P*=0.1911 |  |
|  |  |  | Subject | ns | F(45,180)=1.366, *P*=0.0797 |  |
|  |  | Time(s) | | * | F(4,40)=2.802, *P*=**0.0385** | One-way ANOVA |
|  |  | Number of entries | | * | F(4,40)=2.679, *P*=**0.0453** |  |
| **Fig.5** | **TUNEL** | CA1 | | **** | F(4,15)=71.10, *P*<**0.0001** | One-way ANOVA |
|  |  | CA2 | | **** | F(4,15)=113.1, *P*<**0.0001** |  |
|  |  | CA3 | | **** | F(4,15)=67.21, *P*<**0.0001** |  |
|  |  | DG | | **** | F(4,15)=43.80, *P*<**0.0001** |  |
| **Fig.6** | **ChAT positive cells** | CA1 | | *** | F(4,20)=10.40, *P*=**0.0001** | One-way ANOVA |
|  |  | CA2 | | * | F(4,20)=3.224, *P*=**0.0339** |  |
|  |  | CA3 | | * | F(4,20)=3.219, *P*=**0.0341** |  |
|  |  | DG | | * | F(4,20)=4.195, *P*=**0.0126** |  |
| **Fig.7** | **IHC** | p-p65 | | **** | F(2,9)=54.74, *P*<**0.0001** | One-way ANOVA |
|  |  | VEGFA | | ** | F(1.065,3.196)=87.69, *P*=**0.0019** |  |
|  | **WB** | p-p65/NF-kB/β-actin | | **** | F(2,21)=29.83, *P*<**0.0001** | One-way ANOVA |
|  |  | VEGFA/β-actin | | ** | F(2,21)=8.917, *P*=**0.0016** |  |
|  |  | Occludin/β-actin | | * | F(2,21)=5.703, *P*=**0.0105** |  |
|  |  | ZO-1/β-actin | | ** | F(2,21)=6.459, *P*=**0.0065** |  |
